# Development and Validation of Enzyme-Linked Immunosorbent Assay for Group B Streptococcal Polysaccharide Vaccine

**DOI:** 10.3390/vaccines9060545

**Published:** 2021-05-21

**Authors:** A-Yeung Jang, Min-Joo Choi, Yong Zhi, Hyun-Jung Ji, Ji-Yun Noh, Jin-Gu Yoon, Hee-Jin Cheong, Woo-Joo Kim, Ho-Seong Seo, Joon-Young Song

**Affiliations:** 1 Department of Internal Medicine, Division of Infectious Diseases, Korea University College of Medicine, Seoul 08308, Korea; esooyun@nate.com (A.-Y.J.); jynoh@korea.ac.kr (J.-Y.N.); zephirisj9@gmail.com (J.-G.Y.); heejinmd@korea.ac.kr (H.-J.C.); wjkim@korea.ac.kr (W.-J.K.); 2 Department of Internal Medicine, International St. Mary’s Hospital, Catholic Kwandong University College of Medicine, Incheon 22711, Korea; cowgow@naver.com; 3 Research Division for Radiation Science, Korea Atomic Energy Research Institute, Jeongeup 56212, Korea; yongzhi@kaeri.re.kr (Y.Z.); hyunjung@kaeri.re.kr (H.-J.J.); 4 Department of Radiation Science, University of Science and Technology, Daejeon 34113, Korea; 5 Department of Oral Microbiology and Immunology, DRI and BK21 Plus Program, School of Dentistry, Seoul National University, Seoul 08826, Korea

**Keywords:** *Streptococcus agalactiae*, capsular polysaccharide, enzyme-linked immunosorbent assay, validation, vaccine

## Abstract

*Streptococcus agalactiae* (group B *Streptococcus*, GBS) is a leading cause of neonatal sepsis and meningitis in infants. Limitations of prenatal GBS screening and intrapartum antibiotic prophylaxis render developing GBS vaccines a high priority. In this study, we developed an enzyme-linked immunosorbent assay (ELISA) for the practical and large-scale evaluation of GBS capsular polysaccharide (PS) vaccine immunogenicity against three main serotypes, Ia, III, and V. GBS-ELISA was developed and subsequently validated using a standardized curve-fitting four-parameter logistic method. Specificity was measured using adsorption of serum with homologous and heterologous PS. Homologous adsorption showed a ≥75% inhibition of all three serotypes, whereas with heterologous PS, IgG GBS-ELISA inhibited only ≤25% of serotypes III and V. However, with serotype Ia, IgG antibody levels decreased by >50%, even after adsorption with heterologous PS (III or V). In comparison, the inhibition opsonophagocytic killing assay (OPA) of serotypes Ia GBS exhibited a reduction in opsonophagocytic activity of only 20% and 1.1% for serotypes III and V GBS, respectively. The precision of the GBS-ELISA was assessed in five independent experiments using four serum samples. The coefficient of variation was <5% for all three serotypes. This standardized GBS-ELISA would be useful for GBS vaccine development and its evaluation.

## 1. Introduction

*Streptococcus agalactiae* (group B *Streptococcus*, GBS) is a leading cause of neonatal sepsis and meningitis in infants, resulting in approximately 90,000 infant deaths worldwide annually [1]. Maternal rectovaginal GBS colonization is a major risk factor for preterm birth and invasive disease in newborns [2]. Thus, prenatal GBS culture screening and intrapartum antibiotic prophylaxis (IAP) for high-risk pregnant women have been effective in reducing early onset disease (EOD) [3,4]. However, prenatal screening-based IAP is difficult to implement in resource-limited countries and cannot prevent late-onset disease (LOD). Approximately, 60–80% of LOD is known to occur in infants of mothers with negative prenatal GBS screening at 35 to 37 weeks of gestation, reflecting frequent maternal GBS recolonization and early postnatal acquisition [2]. Several studies have reported a correlation between low maternal serotype-specific anti-capsular GBS antibodies and invasive GBS diseases in newborns [5]. Therefore, maternal GBS vaccination has been proposed as the best alternative strategy to prevent both EOD and LOD. GBS is also associated with a high rate of invasive disease in older adults aged over 65 years and in patients with chronic disease with diabetes, malignancy, liver cirrhosis, and immunocompromising conditions [2]. 

Considering the limitations of the current prenatal screening-based IAP strategy and the increased disease burden of GBS infections in the older population, the development of a GBS vaccine is of high priority [6]. Among several types of GBS vaccines, polysaccharide (PS)-conjugated vaccines (PCVs) have completed phase I/II clinical trials [7,8,9]. The World Health Organization (WHO) Technical Consultation Meeting on GBS vaccines discussed the evaluation of GBS vaccine efficacy in phase III trials due to the low incidence of GBS invasive disease [6,10].

As the maternal capsule antibody threshold has been correlated with clinical protection in recent studies, a GBS vaccine license based on immunogenicity assay has been proposed as an alternative option [11,12]. The Neisseria meningococcal group C conjugate vaccine has been approved based on the results of immunogenicity assays [13]. In this study, we aimed to develop and validate a GBS enzyme-linked immunosorbent assay (GBS-ELISA) to quantify anti-capsular PS-specific antibodies in human serum for the practical and large-scale assessment of GBS vaccine immunogenicity.

## 2. Materials and Methods

### 2.1. Ethics Statement

Among eight pools of sera donated by 25–40 people respectively, four of them were selected considering titers of opsonophagocytic killing assay (OPA): two with high OPA tiers and two with low OPA titers to serotypes Ia, III or V. These four pooled serum samples were used to optimize and validate GBS-ELISA. 

### 2.2. Reagents

All chemical reagents used in this study were purchased from Sigma-Aldrich (St. Louis, MO, USA).

### 2.3. Bacteria Strains

Bacteria used in this study are listed in Table 1. GBS was grown in Todd Hewitt broth (THB; Becton Dickinson, Franklin Lakes, NJ, USA) in a rotary shaker at 100 rpm and at 37 °C. Capsular serotyping was conducted using a Strep-B-Latex kit (Statens Serum Institute, Copenhagen, Denmark). The non-encapsulated isolate (NSP14-358) was confirmed using the Strep-B-Latex kit and whole genome sequencing using PacBio single-molecule sequencers (Macrogen Inc., Seoul, Korea). De novo assembly, genome annotation, and comparative genomic analyses were performed as previously described [14,15,16].

### 2.4. Purification of GBS PS

A 50 mL overnight culture was inoculated into 5 L tryptic soy broth (TSB; MBcell, Seoul, Korea) and shaken at 100 rpm for 12 h at 37 °C. The bacterial cells were recovered by centrifugation and washed twice with phosphate-buffered saline (PBS, pH 7.4). The pellet (approximately 30 g, wet weight) was suspended in 40 mL 2 N sodium hydroxide (NaOH) and 50 g sodium borohydride at 125 rpm for 16 h at 37 °C. The supernatant was recovered following centrifugation and dialyzed against water using a Labscale tangential flow filtration (TFF) system (Millipore, Burlington, MA, USA) equipped with a 30 kDa molecular weight cut-off (MWCO) membrane filter (Millipore; Merck, Darmstadt, Germany) and then concentrated to a minimum volume of approximately 50 mL. The concentrated fractions were re-acetylated by the dropwise addition of acetic anhydride to a final concentration of 0.8 M. The reaction mixture was stirred at room temperature (RT) for 1 h at pH 9 and then the pH was increased to pH 13 by adding 5 N NaOH, followed by stirring for an additional 90 min. The reaction mixture was then adjusted to pH 8 using 6 N hydrochloric acid (HCl). The protein and DNA concentrations of the purified PS were measured using direct ultraviolet (UV) photometry at 260 nm and 280 nm, respectively. The PS concentration was calculated based on the anthrone reaction as described previously [17]. Sialic acid content was determined using a modification of the resorcinol assay, as described previously [18,19]. Briefly, a 0.05 mg/mL sample was dissolved in 1 mL resorcinol reagent (10 mL 2% resorcinol in deionized water [dH_2_O] was added to 80 mL concentrated HCl containing 0.25 mL of 0.1 M copper sulfate (CuSO_4_)). The volume of the reagent was adjusted to 100 mL with dH_2_O. The reagent was prepared at least 4 h before use and was stable for a week when refrigerated at 4 °C. The samples were heated at 110 °C for 25 min, cooled in water for 4 min, and then 2 mL of the hydrolysis solution (butyl acetate: butanol = 85:15, *v*/*v*) was added to each sample. Then, 1 mL of the organic phase of the sample was transferred to a cuvette and read at 580 nm using a UV spectrometer.

### 2.5. PS Immobilization on 96-Well Plate

To compare the stabilization of the immobilization efficiency of PS in 96-well plates, antibody titers were compared following storage after coating of PS (10 μg/mL) the samples on the various types of immunoplates: Maxibinding Immunoplate (SPL, Pocheon, South Korea), fibronectin-coated (SPL), extracellular matrix (ECM)-coated (SPL), collagen type I-coated (SPL), collagen type IV-coated (SPL), and poly D-lysine-coated (SPL) plates. The coated plates were stored at 4 °C for 21 days while maintaining humidity to minimize evaporation of coating components and buffer, followed by GBS-ELISA to measure PS-specific antibody titers. The coated plates were sealed with the sealing tapes and stored in an appropriate humidity (50 ± 5%) box.

To optimize the coating concentration of PS, a range of purified PS (1–10 μg/mL in PBS) was coated on a Maxibinding Immunoplate. Each concentration was assessed in duplicate and compared. For each serotype, the PS coating concentration showing the highest correlation coefficient was selected by evaluating the correlation coefficient between the PS concentration and optical density (OD). The degree of inhibition following adsorption with homologous and heterologous PS was compared.

### 2.6. GBS-ELISA

**Figure 1:**
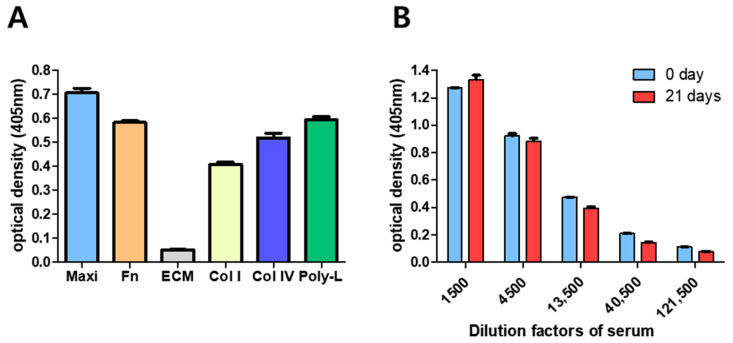
Optimization of group B *Streptococcus* (GBS) polysaccharide (PS) immobilization condition. (**A**) Binding efficiency of GBS Ia PS to different 96-well plates. GBS Ia PS (1 μg/well) was immobilized in indicated 96-well plates at 37 °C for 5 h. Maxi, Maxibinding plate; Fn, fibronectin coated plate; ECM, extracellular matrix (ECM)-coated plate; Col I, collagen type I coated plate; Col IV, collagen type IV coated plate; Poly-L, poly-D-lysine coated plate. (**B**) Comparison of stability of GBS PS in Maxibinding plates for 21 days. GBS Ia PS (1 μg/well) was immobilized in 96-well plates at 37 °C for 5 h and then stored at 4 °C for 21 days. After blocking with 10% fetal bovine serum (FBS), rabbit anti-GBS Ia antiserum (1:5000) was added and bound antiserum was detected using anti-rabbit IgG-horseradish peroxidase (HRP) secondary antibody (1:10,000).

The PS-specific IgG titer was determined according to the pneumococcal ELISA protocol (Figure 1), with minor modifications [20]. Maxibinding immunoplates were coated with 100 μL Ia, III, or V PSs (1 μg/mL) in the PBS and incubated at 37 °C for 5 h. The coated plates were washed three times with PBS containing 0.075% Tween 20 (PBST, Sigma-Aldrich), followed by blocking with 10% fetal bovine serum (FBS) in PBS for 1 h at RT. The pooled sera were adsorbed with the formalin-killed non-encapsulated GBS strain (NSP14-358) for 1 h at RT with gentle shaking. The adsorbed sera were serially 2-fold diluted in PBST on the plates, which were then incubated for 2 h at RT, followed by washing with PBST to remove the unbound antibodies. Goat anti-human IgG-alkaline phosphatase conjugate (1:7500; SouthernBiotech, Birmingham, AL, USA) was added to the wells and incubated for 2 h at RT. The plates were then washed five times with PBST, and 100 µL of AP substrate reagent (1 mg/mL *p*-nitrophenyl phosphate in the diethanolamine substrate buffer) was added to each well. When the color developed, 50 µL of 3 N NaOH was added, and the absorbance was measured at 405 nm (test wavelength) and 690 nm (reference wavelength) using a Spectramax 190 plate reader (Molecular Devices, San Jose, CA, USA). The assigned titer value was indicative of the last dilution with an A_405_ < 0.100.

Data were analyzed using the standardized curve-fit four-parameter logistic method (4-PL) in the pneumococcal PS ELISA assay manual. The respective constant values of the 4-PL equation (f1 = min + (max − min)/{1 + [x/EC_50_]^[Hillslope]}) were determined using pooled sera with reference pooled sera as described previously [21]. Each sample was serially diluted 2-fold, and the OD was measured. The results were analyzed using GraphPad Prism 5 software (GraphPad Software, San Diego, CA, USA). The OD of each sample was applied to the 4-PL equation to calculate the ELISA IgG antibody level (units/mL). The OD (405–690 nm) at a dilution factor of ≤2.0 was compared with the highest concentration of pooled sera and multiplied by the difference to yield the final value.

### 2.7. Validation of GBS-ELISA

The assay was validated for specificity and precision, which was evaluated using four pooled serum samples. The coefficient of variation (CV) was calculated from five independent analyses under the same conditions to evaluate the precision (reproducibility). The specificity of the assay was determined using inhibition ELISA by pre-adsorbing serum samples with heterologous or homologous serotype GBS PS. Data were represented by optical density (405–690 nm) with CV.

### 2.8. Inhibition ELISA

To determine the specificity of the antibody titer, an inhibition ELISA was performed using the homologous or heterologous GBS PS as the inhibitor. Maxibinding immunoplates were coated with 100 μL Ia, III, or V PSs (10 μg/mL) and incubated at 37 °C for 5 h. The coated plates were stored at 4 °C until further use. The pooled serum samples were incubated with homologous or heterologous PS (10 μg/1 mL) for 1 h with gentle shaking. The coated plates were washed three times with PBST, and blocking buffer (10% FBS) was added. After 1 h, pooled serum samples containing the inhibitor were added to the coated plate and incubated for 2 h at RT. Subsequent steps were carried out as described above using GBS-ELISA. Inhibition (%) = (Sample OD/no inhibitor OD) × 100.

### 2.9. Opsonophagocytic Killing Assay

GBS OPA was performed as described previously [21] with minor modifications. To minimize non-specific responses, heat-inactivated pooled serum samples were mixed with 10^8^ CFU of inactivated non-capsulated GBS in PBS (9:1 = *v*/*v*) for 2 h at 4 °C with gentle shaking. Then, the supernatant was harvested by centrifugation (13,000 rpm, 2 min) and the adsorbed pooled serum samples were reacted with antibiotic-resistant GBS Ia, III, and V for 30 min at RT with shaking (700 rpm) on a 96 well plate (SPL). A mixture of differentiated HL60 cells (1 × 10^7^ cells/mL) and complement (4:1) was added to the assay plate and incubated for 45 min at 37 °C with shaking. To stop the reaction, the assay plates were stored on ice for 20 min. Subsequently, 10 μL of the final reaction mixture was spotted onto Todd Hewitt broth supplemented with 1.5% agar (THY agar) with 0.5% yeast extract and overlaid with THY agar (0.5% agar) containing 2,3,4-triphenyltetrazolium chloride (TTC, Sigma-Aldrich, St. Louis, MO, USA). The agar plates were incubated overnight at 37 °C and then the bacterial colonies were counted using NICE software (NIST, Gaithersburg, MD, USA).

### 2.10. Statistical Analysis

All statistical analyses were performed using statistical package for the social sciences (SPSS) version 18.0 (SPSS Korea; Seoul, Republic of Korea). For the correlation between PS concentration and OD, coefficient of determination (r^2^) values ≥ 0.95 in the linear correlation analysis were considered acceptable. For precision, test conditions were considered equivalent and reproducible at a CV (coefficient of variation) ≤20. 

## 3. Results

### 3.1. Optimization of GBS-ELISA 

For the optimization of the developed GBS-ELISA, we first selected the coating plate by comparing several commercial immunoplates, consisting of a Maxibinding immunoplate, fibronectin-coated, ECM-coated, collagen type I-coated, collagen type IV-coated, and poly D-lysine-coated plates. When the plates were coated with the same amount of GBS PS Ia, we found that the Maxibinding plate exhibited the highest OD, which was 13.84 times higher than that of the ECM plate, which showed the lowest binding capacity (Figure 1A).

According to the pneumococcal ELISA protocol [20], the efficiency of the PS coating is expected to be maintained for at least 2 weeks when stored at 4 °C. To confirm the stability of the coating efficiency on the Maxibinding plate, GBS PS Ia was immobilized and anti-PS antibody binding was detected on day 0 and 21 (Figure 1B). Compared with day 0, the binding affinity decreased by approximately 16.4 ± 2.40% on day 21 at a 13,500-fold dilution. Though its stability decreased daily, there was no severe reduction in stability when the GBS-ELISA was compared to the pneumococcal ELISA. There was no significant difference in the non-specific binding of human serum to immobilized GBS PS, following treatment with various blocking reagents such as 0.05% Tween20, 10% FBS, 1% skim milk, 1% casein, or 3% bovine serum albumin (BSA). We chose 10% FBS and PBST as the blocking and washing buffers, respectively because they showed the most reliable results compared to those by others (data not shown).

**Figure 2:**
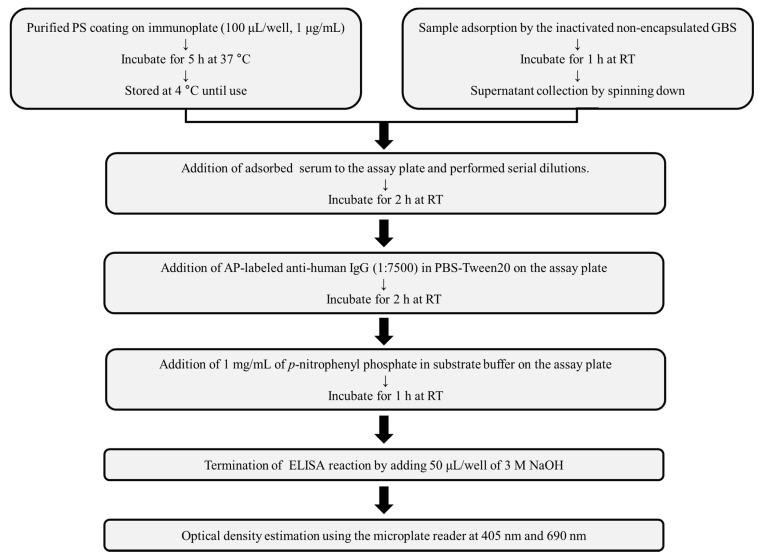
Schematic overview of enzyme-linked immunosorbent assay (ELISA) to measure group B *Streptococcus* (GBS) polysaccharide (PS)-specific IgG. To reduce non-specific binding, samples were reacted with inactivated non-encapsulated GBS before ELISA. Subsequent steps were performed according to the pneumococcal ELISA protocol. PS, polysaccharide; RT, room temperature; AP, alkaline phosphatase.

To optimize the concentration of coated GBS PS for the GBS-ELISA, Maxibinding plates were coated with a range of concentrations of Ia, III, or V GBS PS and the binding of pooled human serum samples collected in a previous study was measured, and the dose-related correlation coefficient was calculated (Table 2) [21]. To find the most optimal PS concentration, we referred to the pneumococcal ELISA protocol, which presented the appropriate PS coating concentration ranging from 1 μg/mL to 10 μg/mL [20]. Thus, we attempted to set the coating concentration at the range of 1–10 μg/mL. The results showed that 1.25–2.5 μg/mL Ia PS, 5–10 μg/mL III, and 1.25–2.5 μg/mL V PS showed r^2^ values of 0.95, 0.99, and 0.98, respectively. Because we confirmed high optical density and r^2^ value at most concentrations ranging from 1 μg/mL to 10 μg/mL, the PS-coating concentration was selected at 1.25μg/mL to measure antibody concentration sufficiently even with low titer sera. Thus, GBS-ELISA was finally optimized as described in Figure 2.

### 3.2. GBS-ELISA Validation

#### 3.2.1. Specificity

**Figure 3:**
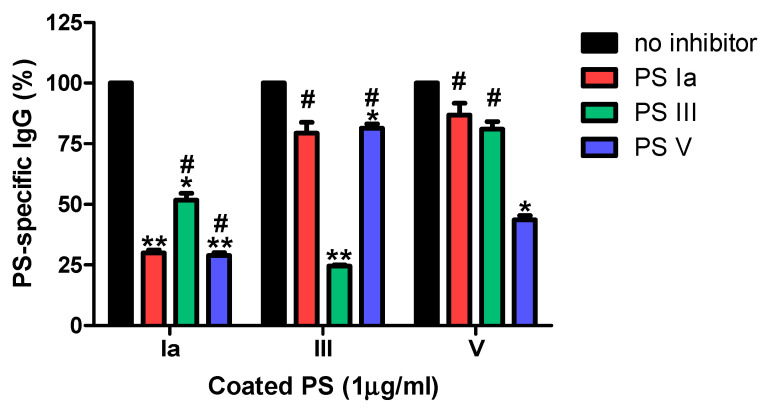
Inhibition group B *Streptococcus* (GBS)-enzyme-linked immunosorbent assay (ELISA) using homologous or heterologous GBS polysaccharide (PS). To confirm specificity of PS-ELISA, pooled serum samples were adsorbed with PS Ia, III or V (10 μg/mL) for 1 h. Black bars (no inhibitor) indicate percentage determined by measuring PS-specific IgG without inhibitor. This assay was conducted in duplicate. Error bars are standard deviations of each condition. * *p* < 0.05, ** *p* < 0.01 compared to the results without inhibition by PS (no inhibitor). # *p* < 0.05 compared to the results with inhibition by homologous PS.

As shown in Figure 3, all three serotype-specific GBS-ELISA procedures showed ≥55% inhibition when adsorbed with the homologous serotype PS.

**Figure 4:**
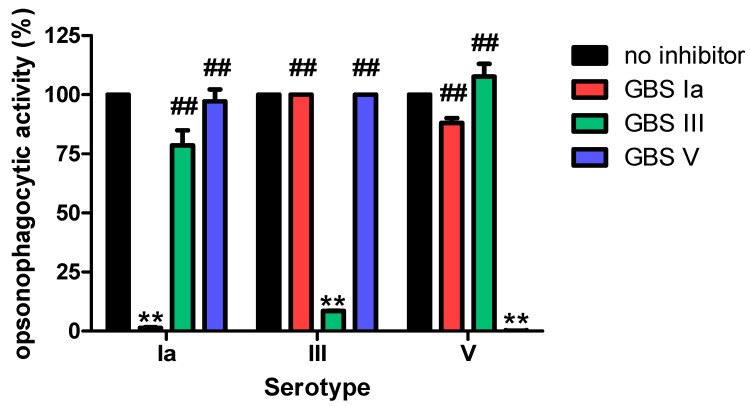
Opsonophagocytic killing assay (OPA) performed by adsorption with inactivated group B *Streptococcus* (GBS) Ia, III, or V in pooled serum samples. This assay was conducted in triplicate. Error bars are standard deviations of each condition. ** *p* < 0.01 compared to the results without inhibition by inactivated GBS (no inhibitor). ## *p* < 0.01 compared to the results with inhibition by homologous serotype inactivated GBS.

When performed with adsorbed heterologous serotype PS, the IgG ELISA showed ≤20% inhibition in the serotype III and V PS GBS-ELISA. However, Ia PS specific IgG antibody levels were decreased by >45% after adsorption with heterologous serotype PS III or V, indicating that antibodies against PS Ia were less specific. To determine if non-specific Ia antibodies are functional, we conducted additional inhibition OPA studies [21]. Contrary to the results of the inhibition ELISA, pre-adsorption of the pooled serum sample with heterologous GBS serotype resulted in an unremarkable reduction in opsonophagocytic activity: 20% and 1.1% adsorption with serotype III and serotype V GBS, respectively (Figure 4). Because non-specific antibody binding can affect the results of GBS-ELISA, it is necessary to reduce or exclude non-specific reactions or reduce errors by comparing GBS-ELISA data with those of the OPA.

#### 3.2.2. Precision (Reproducibility)

Unlike the previous study that measured GBS PS specific antibodies for the large number of samples using Luminex-based multiplex analysis [22], we had a limitation to use just four pooled sera for each PS. Nevertheless, we conducted experiments with selected samples with different OPA titers to validate our GBS-ELISA protocol. The reproducibility of the GBS-ELISA under normal operating conditions (inter-assay variation) was evaluated by calculating the CV of the IgG antibody titer using four pooled serum samples. The CV of the three serotypes was <20% (Table 3) and the average CV values of serotypes Ia, III, and V were 3.3%, 1.9%, and 1.6%, respectively.

## 4. Discussion

In this study, we developed a GBS-ELISA targeting serotypes Ia, III, and V, which caused approximately 81% of EOD and LOD in a systematic meta-analysis [23]. To develop the GBS-ELISA method, PS was purified and the ELISA protocol was optimized to minimize non-specific antibody responses. Subsequently, the specificity and precision were verified. Regarding the accuracy of the assay, a reference standard serum with an established IgG titer for each serotype or another standard test method would allow evaluation of the assay accuracy. However, there are currently no available reference standard sera and established experimental methods to measure the accuracy of GBS-ELISA for GBS vaccines, which is still in its developmental stage. Thus, accuracy validation would be possible when GBS vaccines are developed and reference standard sera are available in the future. Reference standard sera are also required to reduce interlaboratory variability, as described for immunogenicity assays for pneumococcal vaccines [24].

During the optimization process of the GBS-ELISA, the Maxibinding plate was the most efficient for PS coating, followed by poly D-lysine-coated and fibronectin-coated plates. The Maxibinding plate preserved >70% of the PS coating for up to 14 days post-coating. The shelf-life of the PS coatings is important for large-scale immunogenicity evaluations. Maxibinding plates have a modified polystyrene surface with a high binding capacity for molecules with both hydrophilic and hydrophobic regions. 

Previously, pre-adsorption was performed with a non-capsulated GBS strain to prevent the non-specific antibody response during the OPA optimization process [21]. Likewise, in the GBS-ELISA developed in this study, pre-adsorption was performed with a non-capsulated GBS strain. To select the optimal PS coating concentration, various PS-coating concentrations were compared using ELISA. The serum samples were serially diluted for each coating concentration, and the concentration with a stable antibody titer was subsequently selected when the correlation coefficient was maintained above 0.95. For all three serotypes, antibody titer measurements were similar at ≥0.625 μg/mL, and a concentration of 1.25 μg/mL was finally determined to produce data stability and meet the correlation coefficient criteria (≥0.95).

For the specificity validation of the GBS-ELISA, an inhibition ELISA was performed using pooled serum samples from healthy adults. The assay showed good specificity for serotypes III and V, but exhibited non-specific cross-reactive immune response to serotype Ia. IgG antibody levels against serotype Ia appeared to reflect non-specific antibody reactions against serotype III and V PS. According to the inhibition OPA, the functional antibody assay also showed high specificity for serotype Ia. Thus, IgG antibodies against serotypes III and V bind to serotype Ia PS, but may not be functional antibodies that cause opsonophagocytosis. In this study, measurements of IgG antibodies against serotype Ia and other serotypes showed similar levels. However, a previous study showed a significantly lower OPA titer for serotype Ia than that for serotypes III and V [25]. Depending on the serotype, pre-adsorption of the non-capsulated GBS strain would not be sufficient and as in the case of pneumococcus, pre-adsorption using the clinically rare encapsulated GBS strain may be necessary to remove non-specific antibody binding [26].

Because ELISA does not measure the functional capacity of antibodies but rather their capacity to bind to PS immobilized on a plate surface, the correlation between ELISA IgG antibody levels and OPA titers needs to be evaluated. In the DEVANI study conducted in Europe, the correlation between ELISA IgG antibody levels and OPA titers against serotypes Ia, Ib, and III GBS was analyzed using maternal serum samples [27]. The result showed a 70–80% agreement between ELISA IgG antibody levels of 1 µg/mL and OPA titers of 64–128. However, as observed with the pneumococcal immunogenicity assessment, there may be significant age- and serotype-dependent differences in the correlation between ELISA IgG antibody levels and OPA titers [26]. Furthermore, with pneumococcus, there is a low correlation between ELISA and OPA among adults, which is much lower than that observed in children. IgG antibody levels ≥0.35 mg/L and OPA titers ≥1:8 are suggested as protective thresholds for invasive pneumococcal infections in children, but not in adults [26]. In adults, the seroprotective threshold is considered to vary considerably based on pneumococcal serotypes. Serocorrelates of protection are still unclear for invasive GBS infections, even in children. Protective serotype-specific IgG antibody levels for invasive GBS infection have been reported to range from 0.5–10 μg/mL in neonates and infants [19]. However, the seroprotective level varied depending on the serotype and study region.

This study has some limitations. First, reference standard serum was not available and, therefore, the accuracy evaluation of GBNS-ELISA was not feasible. Second, the GBS-ELISA developed in this study only evaluated three main serotypes (Ia, III, and V) among all 10 known GBS serotypes. However, these three serotypes account for >81% of all invasive GBS infections, and this assay could be expanded to other serotypes if necessary [2]. In conclusion, we developed and validated a GBS-ELISA for three main serotypes. Further studies are required to assess the correlation between IgG antibody levels and OPA titers and to establish serocorrelates of protection.

## Figures and Tables

**Table 1:** Group B Streptococcus strains used in this study.

Name	Serotypes	Characteristics	Origin
S10118	Ia	None	Blood
NSP14-212	III	Levofloxacin-resistant	CSF
NSP14-168	V	Erythromycin-resistant	Blood
NSP14-358	Non-typeable	Levofloxacin -resistant	Conjunctiva

**Table 2:** Correlation (coefficient of determination [r^2^]) between dilution factors and absorbance (optical density, OD) according to polysaccharide (PS) coating concentration.

Serotypes	Dilution Fold	PS Concentration (μg/mL)					
		10	5	2.5	1.25	0.625	0
Serotype Ia absorbance (405 nm)	40	3.331	3.240	3.263	3.303	3.316	0.237
	80	3.157	3.026	3.081	3.142	3.179	0.182
	160	2.749	2.550	2.570	2.764	2.821	0.126
	320	2.132	1.934	1.990	2.173	2.274	0.099
	640	1.399	1.222	1.325	1.465	1.436	0.076
	r^2^	0.95	0.97	0.97	0.95	0.92	0.96
Serotype III absorbance (405 nm)	40	2.094	1.933	1.707	1.393	0.987	0.194
	80	1.564	1.432	1.249	1.001	0.696	0.140
	160	1.089	0.970	0.845	0.684	0.474	0.107
	320	0.698	0.635	0.555	0.457	0.309	0.088
	640	0.424	0.396	0.339	0.278	0.201	0.044
	r^2^	0.99	0.98	0.98	0.98	0.97	0.97
Serotype V absorbance (405 nm)	40	2.448	2.319	2.224	2.087	1.844	0.209
	80	1.887	1.751	1.651	1.482	1.256	0.161
	160	1.295	1.189	1.104	0.947	0.782	0.120
	320	0.799	0.727	0.674	0.569	0.451	0.090
	640	0.455	0.409	0.381	0.312	0.248	0.073
	r^2^	0.99	0.99	0.98	0.97	0.96	0.97

**Table 3:** Inter-assay precision of group B *Streptococcus* enzyme-linked immunosorbent assay (GBS-ELISA) for serotype-specific IgG.

Sample	Serotype Ia		Serotype III		Serotype V	
	Mean ± SD	CV (%)	Mean ± SD	CV (%)	Mean ± SD	CV (%)
Sample 1	1.792 ± 0.04	2.3	1.783 ± 0.04	2.0	1.913 ± 0.03	1.7
Sample 2	1.524 ± 0.04	2.5	1.703 ± 0.03	1.5	1.903 ± 0.04	2.2
Sample 3	1.614 ± 0.09	5.3	1.747 ± 0.02	0.9	1.910 ± 0.03	1.5
Sample 4	1.699 ± 0.05	3.2	1.663 ± 0.05	3.2	1.905 ± 0.02	0.9

## Data Availability

Data is contained within the article.
